# Supplementary material for: Middle ratings rise regardless of grammatical construction: Testing syntactic variability in a repeated exposure paradigm
Source: PLoS One. 2021 May 11;16(5):e0251280. doi: 10.1371/journal.pone.0251280 (PMC8112649; doi:10.1371/journal.pone.0251280)
Supplement: S1 Table — (DOCX) [file pone.0251280.s001.docx]

**S1 Table: Fixed effect estimates for secondary LMMs (Experiments 1, 2, and 3)**

─────┼────────────────────────────────────────────────────────────────

Row │ Terms_1 b_1 z_1 Terms_2_3 b_2 z_2 b_3 z_3

─────┼────────────────────────────────────────────────────────────────

**Mean of targets**

1 │ wer_wen 4.90 29.66 SO_wh 5.45 33.27 5.85 47.93

2 │ wer_was 4.81 30.33 SO_which 5.27 31.45 5.59 43.23

3 │ wen_wer 4.01 25.86 OS_wh 3.36 21.87 3.26 18.01

4 │ was_wer 3.68 22.30 OS_which 4.87 27.28 5.30 35.76

**Delta of targets**

5 │ ∆-wer_wen 0.00 0.03 b2SO_wh -0.08 -0.78 0.34 2.42

6 │ ∆-wer_was 0.44 3.31 b2SO_which -0.19 -1.33 0.17 1.34

7 │ ∆-wen_wer 0.47 3.45 b2OS_wh 0.58 4.46 0.44 3.70

8 │ ∆-was_wer 0.31 2.11 b2OS_which 0.32 2.52 0.57 3.76

**Mean of fillers**

9 │ A 6.55 46.58 A 6.40 56.70 6.56 75.59

10 │ B 5.88 40.19 B 5.66 34.12 5.68 37.56

11 │ C 4.57 31.79 C 3.99 25.10 4.31 26.51

12 │ D 3.04 21.40 D 3.14 19.84 3.29 21.49

13 │ E 2.22 16.54 E 2.10 15.70 1.97 15.13

14 │ F 1.59 11.85 F 1.82 15.55 1.73 15.27

**Delta of fillers**

15 │ ∆-A 0.09 1.34 b2A -0.13 -1.35 0.03 0.29

16 │ ∆-B 0.19 1.82 b2B 0.09 0.84 0.40 3.16

17 │ ∆-C 0.59 4.00 b2C 0.46 3.96 0.34 2.39

18 │ ∆-D 0.13 0.89 b2D 0.42 3.61 0.46 4.60

19 │ ∆-E 0.37 4.43 b2E 0.40 4.72 0.25 3.62

20 │ ∆-F -0.13 -1.52 b2F 0.01 0.15 -0.06 -0.65

─────┼────────────────────────────────────────────────────────────────

Note. b_ are estimates for acceptability rating averaged across blocks 2 to 6 for target (rows 1 to 4) and filler (rows 9 to 14) types and the change from block 1 for targets (∆-rows 5 to 8) and fillers (∆-rows 15 to 20); z_ are corresponding z-values for b_1; _1, _2, _3 refer to experiments 1 to 3. The corresponding observed means are shown in Figures 1b, 2b, and 3b.
