# Supplementary material for: Middle ratings rise regardless of grammatical construction: Testing syntactic variability in a repeated exposure paradigm
Source: PLoS One. 2021 May 11;16(5):e0251280. doi: 10.1371/journal.pone.0251280 (PMC8112649; doi:10.1371/journal.pone.0251280)
Supplement: S2 Table — (DOCX) [file pone.0251280.s002.docx]

**S2 Table: Experiment 1 – German sentences (lab):**

**Primary LMM fixed-effect estimates**

|  | **Complex LMM** | | | **Zero-correlation LMM** | | |
| --- | --- | --- | --- | --- | --- | --- |
| *Predictors* | *beta* | *CI* | *z* | *beta* | *CI* | *z* |
| **Grand mean** | 4.30 | 4.03 – 4.57 | **30.85** | 4.30 | 4.03 – 4.57 | **30.85** |
| **Order (so)** | 0.52 | 0.41 – 0.63 | **8.97** | 0.52 | 0.41 – 0.63 | **8.93** |
| Animacy (an) | -0.05 | -0.11 – 0.01 | -1.62 | -0.05 | -0.11 – 0.01 | -1.60 |
| **Block [2-6] – 1 (b1)** | 0.31 | 0.12 – 0.49 | **3.24** | 0.31 | 0.12 – 0.49 | **3.27** |
| Block [3-6] – 2 (b2) | -0.08 | -0.16 – -0.00 | -1.98 | -0.08 | -0.16 – -0.00 | -1.97 |
| Block [4-6] – 3 (b3) | -0.03 | -0.12 – 0.05 | -0.82 | -0.03 | -0.12 – 0.05 | -0.82 |
| Block [5-6] – 4 (b4) | -0.00 | -0.09 – 0.08 | -0.11 | -0.00 | -0.09 – 0.08 | -0.11 |
| Block [6] – 5 (b5) | -0.01 | -0.11 – 0.09 | -0.22 | -0.01 | -0.11 – 0.09 | -0.22 |
| **so x an** | 0.13 | 0.08 – 0.18 | **5.00** | 0.13 | 0.08 – 0.18 | **5.10** |
| so x b1 | -0.08 | -0.19 – 0.02 | -1.58 | -0.08 | -0.19 – 0.02 | -1.59 |
| so x b2 | -0.04 | -0.12 – 0.04 | -1.01 | -0.04 | -0.12 – 0.04 | -1.01 |
| so x b3 | 0.05 | -0.04 – 0.13 | 1.09 | 0.05 | -0.04 – 0.13 | 1.09 |
| so x b4 | -0.01 | -0.10 – 0.08 | -0.22 | -0.01 | -0.10 – 0.08 | -0.22 |
| so x b5 | 0.07 | -0.03 – 0.17 | 1.36 | 0.07 | -0.03 – 0.17 | 1.36 |
| an x b1 | -0.07 | -0.17 – 0.03 | -1.45 | -0.07 | -0.17 – 0.02 | -1.48 |
| an x b2 | -0.04 | -0.12 – 0.04 | -0.92 | -0.04 | -0.12 – 0.04 | -0.92 |
| an x b3 | 0.04 | -0.04 – 0.12 | 1.00 | 0.04 | -0.04 – 0.12 | 1.00 |
| an x b4 | -0.06 | -0.14 – 0.03 | -1.26 | -0.06 | -0.14 – 0.03 | -1.25 |
| an x b5 | 0.03 | -0.07 – 0.13 | 0.63 | 0.03 | -0.07 – 0.13 | 0.63 |
| **(so x an) x b1** | -0.15 | -0.26 – -0.04 | **-2.60** | -0.15 | -0.26 – -0.03 | **-2.55** |
| (so x an) x b2 | -0.02 | -0.10 – 0.06 | -0.55 | -0.02 | -0.10 – 0.06 | -0.55 |
| (so x an) x b3 | 0.04 | -0.04 – 0.12 | 0.96 | 0.04 | -0.04 – 0.12 | 0.95 |
| **(so x an) x b4** | 0.10 | 0.02 – 0.19 | **2.34** | 0.10 | 0.02 – 0.19 | **2.33** |
| (so x an) x b5 | -0.08 | -0.18 – 0.02 | -1.61 | -0.08 | -0.18 – 0.02 | -1.60 |
