# Supplementary material for: Middle ratings rise regardless of grammatical construction: Testing syntactic variability in a repeated exposure paradigm
Source: PLoS One. 2021 May 11;16(5):e0251280. doi: 10.1371/journal.pone.0251280 (PMC8112649; doi:10.1371/journal.pone.0251280)
Supplement: S3 Table — (DOCX) [file pone.0251280.s003.docx]

**S3 Table: Experiment 1 – German sentences (lab):**

**LMM goodness of fit statistics and estimates of parameters**

**Goodness of fit statistics**

Row │ dof deviance AIC AICc BIC

─────┼────────────────────────────────────────────

1 │ 56 56894.1 57006.1 57006.5 57442.3

2 │ 61 56893.8 57015.8 57016.2 57491.0

**3 │ 89 56522.9 56700.9 56701.8 57394.2**

4 │ 107 56480.9 56694.9 56696.2 57528.5

5 │ 209 56160.4 56578.4 56583.4 58206.5

6 │ 345 55970.4 56660.4 56674.0 59348.0

7 │ 441 55927.2 56809.2 56831.6 60244.7

Note. Goodness of fit statistics for selected model (**#3**) and hierarchically nested alternative LMMs. Selection was based on BIC (i.e., lowest value).

**Estimates of model parameters**

| Terms | Est. | SE | z | p | σ_Item | σ_Subj |

|:--------- | -------:| ------:| -----:| ------:| ------:| ------:|

| wer_wen | 4.8975 | 0.1651 | 29.66 | <1e-99 | 0.3326 | 1.1031 |

| wer_was | 4.8133 | 0.1587 | 30.33 | <1e-99 | 0.4092 | 1.0458 |

| wen_wer | 4.0100 | 0.1551 | 25.85 | <1e-99 | 0.3879 | 1.0230 |

| was_wer | 3.6775 | 0.1649 | 22.30 | <1e-99 | 0.2589 | 1.1093 |

| ∆-wer_wen | 0.0017 | 0.1031 | 0.02 | 0.9871 | 0.3723 | 0.4095 |

| ∆-wer_was | 0.4425 | 0.1338 | 3.31 | 0.0009 | 0.6822 | 0.6208 |

| ∆-wen_wer | 0.4650 | 0.1349 | 3.45 | 0.0006 | 0.3189 | 0.7386 |

| ∆-was_wer | 0.3142 | 0.1490 | 2.11 | 0.0350 | 0.4788 | 0.8289 |

| A | 6.5518 | 0.1407 | 46.58 | <1e-99 | 0.4045 | 0.8534 |

| B | 5.8780 | 0.1463 | 40.19 | <1e-99 | 0.4321 | 0.8828 |

| C | 4.5667 | 0.1437 | 31.79 | <1e-99 | 0.4608 | 0.8448 |

| D | 3.0423 | 0.1421 | 21.40 | <1e-99 | 0.3073 | 0.9097 |

| E | 2.2196 | 0.1342 | 16.54 | <1e-60 | 0.3547 | 0.8287 |

| F | 1.5923 | 0.1344 | 11.85 | <1e-31 | 0.3183 | 0.8464 |

| ∆-A | 0.0875 | 0.0654 | 1.34 | 0.1810 | | |

| ∆-B | 0.1935 | 0.1064 | 1.82 | 0.0690 | | 0.5811 |

| ∆-C | 0.5875 | 0.1471 | 4.00 | <1e-04 | 0.4965 | 0.7423 |

| ∆-D | 0.1256 | 0.1405 | 0.89 | 0.3714 | 0.3485 | 0.7768 |

| ∆-E | 0.3685 | 0.0831 | 4.43 | <1e-05 | | 0.3552 |

| ∆-F | -0.1250 | 0.0822 | -1.52 | 0.1282 | 0.3224 | |

| Residual | 1.0945 | | | | | |

Note. Estimates are mean acceptability for the average of blocks 2 to 6; ∆ is change between block 1 and average of blocks 2 to 6. Correlation parameters are not shown.
