# Supplementary material for: Middle ratings rise regardless of grammatical construction: Testing syntactic variability in a repeated exposure paradigm
Source: PLoS One. 2021 May 11;16(5):e0251280. doi: 10.1371/journal.pone.0251280 (PMC8112649; doi:10.1371/journal.pone.0251280)
Supplement: S4 Table — (DOCX) [file pone.0251280.s004.docx]

**S4 Table: Experiment 2 – English sentences (web):**

**Primary LMM fixed-effect estimates**

|  | **Complex LMM** | | | | **Zero-correlation LMM** | | |
| --- | --- | --- | --- | --- | --- | --- | --- |
| *Predictors* | *beta* | *CI* | *z* | *beta* | | *CI* | *z* |
| **Grand mean** | 4.71 | 4.45 – 4.97 | **35.53** | 4.71 | | 4.45 – 4.97 | **35.54** |
| **Order (so)** | 0.67 | 0.56 – 0.78 | **12.20** | 0.67 | | 0.56 – 0.78 | **12.26** |
| **D-linking (dl)** | -0.35 | -0.45 – -0.25 | **-6.74** | -0.35 | | -0.45 – -0.25 | **-6.74** |
| Block [2-6] – 1 (b1) | 0.16 | -0.02 – 0.33 | 1.78 | 0.16 | | -0.02 – 0.33 | 1.77 |
| Block [3-6] – 2 (b2) | -0.08 | -0.17 – 0.00 | -1.91 | -0.08 | | -0.17 – 0.00 | -1.90 |
| **Block [4-6] – 3 (b3)** | **-0.11** | **-0.20 – -0.02** | **-2.46** | -0.11 | | -0.20 – -0.02 | **-2.46** |
| Block [5-6] – 4 (b4) | -0.03 | -0.12 – 0.06 | -0.67 | -0.03 | | -0.12 – 0.06 | -0.67 |
| Block [6] – 5 (b5) | -0.02 | -0.12 – 0.09 | -0.29 | -0.02 | | -0.12 – 0.09 | -0.29 |
| **so x dl** | 0.43 | 0.34 – 0.52 | **9.46** | 0.43 | | 0.34 – 0.52 | **9.52** |
| **so x b1** | -0.29 | -0.41 – -0.18 | **-4.92** | -0.29 | | -0.41 – -0.18 | **-5.01** |
| **so x b2** | -0.14 | -0.22 – -0.05 | **-3.16** | -0.14 | | -0.22 – -0.05 | **-3.16** |
| so x b3 | -0.08 | -0.17 – 0.00 | -1.90 | -0.08 | | -0.17 – 0.00 | -1.90 |
| so x b4 | -0.05 | -0.15 – 0.04 | -1.13 | -0.05 | | -0.15 – 0.04 | -1.13 |
| so x b5 | -0.03 | -0.14 – 0.07 | -0.63 | -0.03 | | -0.14 – 0.07 | -0.63 |
| dl x b1 | 0.09 | -0.01 – 0.19 | 1.76 | 0.09 | | -0.01 – 0.19 | 1.79 |
| **dl x b2** | 0.10 | 0.01 – 0.18 | **2.27** | 0.10 | | 0.01 – 0.18 | **2.27** |
| dl x b3 | 0.01 | -0.08 – 0.10 | 0.19 | 0.01 | | -0.08 – 0.10 | 0.19 |
| dl x b4 | 0.02 | -0.08 – 0.11 | 0.36 | 0.02 | | -0.08 – 0.11 | 0.36 |
| dl x b5 | -0.01 | -0.12 – 0.10 | -0.17 | -0.01 | | -0.12 – 0.10 | -0.17 |
| (so x dl) x b1 | -0.04 | -0.12 – 0.05 | -0.89 | -0.04 | | -0.13 – 0.06 | -0.78 |
| (so x dl) x b2 | -0.02 | -0.11 – 0.06 | -0.58 | -0.02 | | -0.11 – 0.06 | -0.58 |
| (so x dl) x b3 | 0.04 | -0.05 – 0.12 | 0.81 | 0.04 | | -0.05 – 0.12 | 0.81 |
| (so x dl) x b4 | 0.02 | -0.07 – 0.12 | 0.52 | 0.02 | | -0.07 – 0.12 | 0.52 |
| (so x dl) x b5 | 0.03 | -0.08 – 0.14 | 0.55 | 0.03 | | -0.08 – 0.14 | 0.55 |
