# Supplementary material for: Middle ratings rise regardless of grammatical construction: Testing syntactic variability in a repeated exposure paradigm
Source: PLoS One. 2021 May 11;16(5):e0251280. doi: 10.1371/journal.pone.0251280 (PMC8112649; doi:10.1371/journal.pone.0251280)
Supplement: S5 Table — (DOCX) [file pone.0251280.s005.docx]

**S5 Table: Experiment 2 – English sentences (web):**

**Secondary LMM goodness of fit statistics and parameter estimates**

**Goodness of fit statistics**

Row │ dof deviance AIC AICc BIC

─────┼────────────────────────────────────────────

1 │ 54 59256.0 59364.0 59364.3 59784.7

2 │ 61 59249.3 59371.3 59371.7 59846.5

**3 │ 76 59010.3 59162.3 59163.0 59754.3**

4 │ 96 58939.8 59131.8 59132.9 59879.7

5 │ 244 58385.4 58873.4 58880.2 60774.1

6 │ 322 58296.7 58940.7 58952.5 61449.1

7 │ 441 58252.7 59134.7 59157.1 62570.1

Note. Goodness of fit statistics for selected model (**#3**) and hierarchically nested alternative LMMs. Selection was based on BIC (i.e., lowest value).

**Estimates of model parameters**

| Terms | Est. | SE | z | p | σ_Item | σ_Subj |

|:---------- | -------:| ------:| -----:| ------:| ------:| ------:|

| SO_wh | 5.4542 | 0.1639 | 33.27 | <1e-99 | 0.1980 | 1.1035 |

| SO_which | 5.2708 | 0.1676 | 31.45 | <1e-99 | | 1.1367 |

| OS_wh | 3.3625 | 0.1538 | 21.87 | <1e-99 | 0.2548 | 1.0258 |

| OS_which | 4.8742 | 0.1787 | 27.28 | <1e-99 | 0.1731 | 1.2101 |

| ∆-SO_wh | -0.0833 | 0.1067 | -0.78 | 0.4349 | | 0.4561 |

| ∆-SO_which | -0.1875 | 0.1412 | -1.33 | 0.1843 | 0.4361 | 0.7365 |

| ∆-OS_wh | 0.5792 | 0.1299 | 4.46 | <1e-05 | 0.5880 | 0.5771 |

| ∆-OS_which | 0.3242 | 0.1285 | 2.52 | 0.0117 | 0.4180 | 0.6198 |

| A | 6.4024 | 0.1129 | 56.70 | <1e-99 | | 0.7561 |

| B | 5.6631 | 0.1660 | 34.12 | <1e-99 | 0.6390 | 0.9029 |

| C | 3.9929 | 0.1591 | 25.10 | <1e-99 | 0.5245 | 0.9274 |

| D | 3.1399 | 0.1582 | 19.84 | <1e-86 | 0.5395 | 0.9105 |

| E | 2.0970 | 0.1336 | 15.70 | <1e-54 | | 0.9032 |

| F | 1.8185 | 0.1169 | 15.55 | <1e-53 | 0.1765 | 0.7619 |

| ∆-A | -0.1274 | 0.0945 | -1.35 | 0.1775 | | 0.4316 |

| ∆-B | 0.0887 | 0.1058 | 0.84 | 0.4018 | 0.3262 | 0.4166 |

| ∆-C | 0.4571 | 0.1155 | 3.96 | <1e-04 | 0.3771 | 0.4862 |

| ∆-D | 0.4196 | 0.1163 | 3.61 | 0.0003 | 0.2829 | 0.5621 |

| ∆-E | 0.4036 | 0.0854 | 4.72 | <1e-05 | | 0.3289 |

| ∆-F | 0.0149 | 0.0985 | 0.15 | 0.8799 | | 0.4731 |

| Residual | 1.1882 | | | | | |

Note. Estimates are mean acceptability for the average of blocks 2 to 6; ∆ is change between block 1 and average of blocks 2 to 6. Correlation parameters are not shown.
