# Supplementary material for: Middle ratings rise regardless of grammatical construction: Testing syntactic variability in a repeated exposure paradigm
Source: PLoS One. 2021 May 11;16(5):e0251280. doi: 10.1371/journal.pone.0251280 (PMC8112649; doi:10.1371/journal.pone.0251280)
Supplement: S6 Table — (DOCX) [file pone.0251280.s006.docx]

**S6 Table: Experiment 3 – English sentences (lab):**

**Primary LMM fixed-effect estimates**

|  | **Complex LMM** | | | **Zero-correlation LMM** | | |
| --- | --- | --- | --- | --- | --- | --- |
| *Predictors* | *beta* | *CI* | *z* | *beta* | *CI* | *z* |
| **Grand mean** | 4.93 | 4.72 – 5.15 | **44.48** | 4.94 | 4.72 – 5.15 | **44.33** |
| **Order (so)** | 0.74 | 0.63 – 0.85 | **12.93** | 0.74 | 0.62 – 0.85 | **12.81** |
| **D-linking (dl)** | -0.45 | -0.55 – -0.35 | **-8.87** | -0.45 | -0.55 – -0.35 | **-8.92** |
| **Block [2-6] – 1 (b1)** | 0.39 | 0.22 – 0.56 | **4.54** | 0.38 | 0.21 – 0.56 | **4.35** |
| **Block [3-6] – 2 (b2)** | 0.14 | 0.06 – 0.22 | **3.53** | 0.14 | 0.06 – 0.22 | **3.49** |
| Block [4-6] – 3 (b3) | 0.03 | -0.05 – 0.11 | 0.74 | 0.03 | -0.05 – 0.11 | 0.67 |
| Block [5-6] – 4 (b4) | 0.01 | -0.08 – 0.10 | 0.24 | 0.01 | -0.08 – 0.10 | 0.22 |
| Block [6] – 5 (b5) | 0.05 | -0.05 – 0.15 | 1.06 | 0.05 | -0.05 – 0.15 | 1.02 |
| **so x dl** | 0.56 | 0.46 – 0.66 | **10.97** | 0.56 | 0.46 – 0.66 | **11.02** |
| **so x b1** | -0.13 | -0.24 – -0.02 | **-2.24** | -0.12 | -0.23 – -0.01 | **-2.13** |
| **so x b2** | -0.12 | -0.20 – -0.05 | **-3.09** | -0.13 | -0.21 – -0.05 | **-3.17** |
| so x b3 | -0.08 | -0.16 – 0.00 | -1.92 | -0.08 | -0.16 – 0.00 | -1.87 |
| so x b4 | 0.02 | -0.07 – 0.10 | 0.37 | 0.02 | -0.07 – 0.10 | 0.40 |
| so x b5 | 0.05 | -0.05 – 0.15 | 0.93 | 0.05 | -0.05 – 0.15 | 1.03 |
| dl x b1 | 0.01 | -0.11 – 0.13 | 0.17 | 0.01 | -0.11 – 0.13 | 0.16 |
| dl x b2 | 0.07 | -0.01 – 0.14 | 1.66 | 0.07 | -0.01 – 0.14 | 1.62 |
| dl x b3 | 0.03 | -0.05 – 0.11 | 0.72 | 0.04 | -0.04 – 0.13 | 1.05 |
| dl x b4 | 0.00 | -0.08 – 0.09 | 0.02 | 0.01 | -0.07 – 0.10 | 0.29 |
| dl x b5 | 0.06 | -0.04 – 0.16 | 1.14 | 0.07 | -0.03 – 0.17 | 1.36 |
| (so x dl) x b1 | 0.10 | -0.03 – 0.22 | 1.51 | 0.08 | -0.05 – 0.20 | 1.19 |
| (so x dl) x b2 | -0.02 | -0.10 – 0.06 | -0.53 | -0.02 | -0.10 – 0.06 | -0.57 |
| (so x dl) x b3 | -0.01 | -0.09 – 0.07 | -0.22 | 0.00 | -0.08 – 0.09 | 0.09 |
| (so x dl) x b4 | -0.02 | -0.11 – 0.07 | -0.47 | -0.00 | -0.09 – 0.08 | -0.10 |
| (so x dl) x b5 | 0.01 | -0.09 – 0.11 | 0.18 | 0.02 | -0.08 – 0.12 | 0.41 |
