# Supplementary material for: Middle ratings rise regardless of grammatical construction: Testing syntactic variability in a repeated exposure paradigm
Source: PLoS One. 2021 May 11;16(5):e0251280. doi: 10.1371/journal.pone.0251280 (PMC8112649; doi:10.1371/journal.pone.0251280)
Supplement: S7 Table — (DOCX) [file pone.0251280.s007.docx]

**S7 Table: Experiment 3 – English sentences (lab):
Secondary LMM goodness of fit statistics and parameter estimates**

**Goodness of fit statistics**

Row │ dof deviance AIC AICc BIC

─────┼────────────────────────────────────────────

1 │ 53 58009.2 58115.2 58115.6 58528.1

2 │ 61 58004.5 58126.5 58127.0 58601.7

**3 │ 75 57785.6 57935.6 57936.2 58519.8**

4 │ 95 57738.6 57928.6 57929.6 58668.7

5 │ 224 57280.8 57728.8 57734.5 59473.8

6 │ 302 57161.4 57765.4 57775.8 60118.0

7 │ 441 57095.2 57977.2 57999.6 61412.7

Note. Goodness of fit statistics for **selected LMM (#3)** and hierarchically nested alternative LMMs. Selection was based on BIC (i.e., lowest value).

**Estimates of model parameters for LMM #3**

| Terms | Est. | SE | z | p | σ_Item | σ_Subj |

|:---------- | -------:| ------:| -----:| ------:| ------:| ------:|

| SO_wh | 5.8456 | 0.1220 | 47.93 | <1e-99 | 0.1228 | 0.8093 |

| SO_which | 5.5882 | 0.1293 | 43.22 | <1e-99 | | 0.8656 |

| OS_wh | 3.2602 | 0.1811 | 18.01 | <1e-71 | 0.1716 | 1.2284 |

| OS_which | 5.3041 | 0.1483 | 35.76 | <1e-99 | 0.2261 | 0.9913 |

| ∆-SO_wh | 0.3405 | 0.1409 | 2.42 | 0.0157 | | 0.7700 |

| ∆-SO_which | 0.1709 | 0.1279 | 1.34 | 0.1816 | 0.4340 | 0.6006 |

| ∆-OS_wh | 0.4397 | 0.1189 | 3.70 | 0.0002 | 0.3924 | 0.5376 |

| ∆-OS_which | 0.5654 | 0.1503 | 3.76 | 0.0002 | 0.6142 | 0.7460 |

| A | 6.5643 | 0.0868 | 75.59 | <1e-99 | | 0.5695 |

| B | 5.6798 | 0.1512 | 37.56 | <1e-99 | 0.6980 | 0.7095 |

| C | 4.3089 | 0.1625 | 26.51 | <1e-99 | 0.5401 | 0.9473 |

| D | 3.2917 | 0.1532 | 21.49 | <1e-99 | 0.5372 | 0.8713 |

| E | 1.9744 | 0.1305 | 15.13 | <1e-51 | | 0.8829 |

| F | 1.7315 | 0.1134 | 15.27 | <1e-51 | 0.1557 | 0.7428 |

| ∆-A | 0.0345 | 0.1180 | 0.29 | 0.7699 | | 0.6654 |

| ∆-B | 0.3970 | 0.1257 | 3.16 | 0.0016 | 0.3331 | 0.6374 |

| ∆-C | 0.3387 | 0.1419 | 2.39 | 0.0170 | 0.4274 | 0.7297 |

| ∆-D | 0.4583 | 0.0996 | 4.60 | <1e-05 | 0.3894 | 0.2773 |

| ∆-E | 0.2482 | 0.0686 | 3.62 | 0.0003 | | |

| ∆-F | -0.0631 | 0.0975 | -0.65 | 0.5173 | | 0.4797 |

| Residual | 1.1475 | | | | | |

Note. Estimates are mean acceptability for the average of blocks 2 to 6; ∆ is change between block 1 and average of blocks 2 to 6. Correlation parameters are not shown.
