# Supplementary material for: Middle ratings rise regardless of grammatical construction: Testing syntactic variability in a repeated exposure paradigm
Source: PLoS One. 2021 May 11;16(5):e0251280. doi: 10.1371/journal.pone.0251280 (PMC8112649; doi:10.1371/journal.pone.0251280)
Supplement: Attachment — Submitted filename: REVIEW.pdf [file pone.0251280.s008.pdf]

REVIEW OF
Middle ratings rise regardless of grammatical construction: Testing syntactic variability in a new repeated exposure paradigm

The paper presents the results from three graded acceptability judgment surveys on multiple question sentences and superiority, one on German, two on English. The design involves balanced and randomized exposure of the same sentence types over six blocks with pauses in between. Overall, the results show that acceptability ratings slightly increase under repeated exposure, and that speakers prefer subject-initial sentences (Who saw what?) over object-initial ones (What did who see?). Furthermore, subject-object order interacts with animacy in German and D-linking in English making object-subject order more acceptable if an animate or D-linked object precedes and inanimate or non-D-linked subject. The judgments on the four target sentence types in all three experiments fall in the middle range of the 7-point scale (app. 3-5, 3-5.5, 3-6), and the set of 6 different filler types span almost the whole scale (app. 1.5-6.5). As the fillers that are rated highest (type A) and the ones rated lowest (type F) do not increase in acceptability between the first block and the average value for blocks 2-5, it is argued that “satiation” affects a “zone of well-formedness” (i.e., the middle range of acceptability) rather than specific types of constructions. This is seen as going against the assumption in much the literature on processing and acceptability, namely that satiation only affects grammatical sentences.

The study is very interesting and important as it informs an ongoing debate about language processing, parsing, and grammaticality vs. acceptability. I also agree that it would be nice to have a proper standardized experimental design to test for effects of repeated measures (the literature is mixed and some use a binary scale some a continuous one). However, the paper could do with a serious revision and it has a large number of unresolved issues. For example, it is not explained what the zone of well-formedness, and it is not clear how the results go against what is actually argued in the literature. Also, some arguments are based on comparisons between controlled target sentences and mean values for conflated sets of uncontrolled fillers; how this licensed needs to be explained. Some sections are almost impossible to understand, and there are many unexplained terms.

I recommend major revision.

<u>Detailed comments:</u>

P. 4:
Line 86 and 89 (and in many other places): There are references to numbered sections (e.g., 1.1 and 1.2), but the section headings are not numbered.

P. 8:
Line 150: “In Norwegian…” ➔ “In Danish…” The study in ref. [25] is about Danish, not Norwegian.

P. 9:
Line 196 & 199. Please explain that “zone of well-formedness” (on p. 4, line 98-99, it’s called “intermediate zone of judgments”) refers to the middle-range of acceptability on a

continuous scale, and it would also be helpful (and appropriate) if it was specified exactly what is meant by “zone” (is it 2-6 on the scale from 1 to 7?).

P. 10, line 202: “Unfortunately, many studies do not report means.”
Are you sure? The study in [25] clearly does (p. 57: table 2 and fig. 2, p. 63: table 4, p. 64: fig. 4), and the same goes for [17], p. 335: table 3, p. 336: table 4.

Line 204-212: What is scale used for the reported means? They are uninterpretable with this information.

Line 218-219: I don’t understand this sentence. Please reformulate.

Line 221: “already in the 80s, reports were made movement amongst intermediate judgments [6]”
This sentence does not make sense. Please reformulate.

Line 225: “The mean ratings are given in (3), along with their status with regard to well-formedness.” (a) What is the scale behind the means? (b) The is not indication of their well-formedness (apart from the means).

P. 12:
Line 261: “Keeping a block design allows principled points of measurement…”
Why? Testing for the effect of repeated exposure with a high number of tokens per type across a single experiment can just as easily control for individual variation, so why is this proposal better?

Line 272-273: “…condition in (3)…given in (4):” ➔ “…condition in (4)…given in (5):”

P. 13:
Line 276-287: I think the phenomenon could be explained clearer with a more recent formulation. As it stands, it is almost completely opaque to readers not familiar with the original framework. Also, nothing in this paper hinges on this particular definition of the Superiority Condition. It could just as well have been relativized Minimality, Shortest Move, the Minimal Link Condition, Economy – or simply Locality.

Line 288:
”In the sentences in (3), the subject who is superior to (the object what, but …” ➔
”In the sentences in (5), the subject who is superior to (the object what), but …”
(Note also the bracket after “what”.)

Line 289-290: “… (3a) … (3b) …” ➔ “… (5a) … (5b) …”

Line 292-295: Please explain why you think that “a variability with multiple factors” makes superiority violations (or any other construction) “particularly suitable”.

P. 296:
Line 306: I haven’t come across the term “yoking” before. It seems to be an idiosyncratic term used in [31]. Please explain.

P. 15:

Line 330: "… ratings from "1" to "7"…"
Did you label the points on the scale? That seems to be hinted at in lines 382-384 below.

Line 334-335: "Each target sentence was available in four conditions." That cannot be true. That would be 120*4 sentences. Do you mean that the same scenario / argument structure was presented in four different conditions/sentence types?

P. 16:
Are conditions 3 and 4 generally considered ungrammatical or unacceptable in German?

P. 17:
"…meaning that superiority is violated."
Which is normally unacceptable in German, or?

Line 377: "One half of the target sentences had, as in the example above…" Which example?

Line 377-378: "… an adverb at the beginning of the main clause and the other half at the end of the main clause." Why this variation? Please give examples of both.

Line 379-381: There's a verb missing…
"… because the goal of stimulus construction WAS to create …"

Line 381: "and it was certain that the use of adverbs would contribute significantly to this." How can that be certain? Maybe you should say "assumed" instead.

Line 382 (and elsewhere): "distractor" ➔ "filler". Throughout you use the term "filler" far more often than "distractor".

Line 382-383: "For the first five gradations from (A) "almost not well-formed" to (E) "completely well-formed", …"
(i) This seems to be in the wrong order: It should be (A) "complete wellformed" and (E) "almost not wellformed". (ii) This is not the rating terms used in (7) below, where (A) is defined as "Interpretable and highly acceptable", and (E) is "Interpretable but less acceptable than (D)". (iii) There should be an explanation that these labels refer to numerical values on the 7-point scale.

Line 386-387: "We then added a sixth level (F)". Since you use a 7-point scale (line 330), why do you use only 6 filler types if you want the set of fillers to "better reflect the spectrum of grammaticality"? And how do you know that it actually does reflect the full spectrum? Did you check? Did you do a norming study?

P. 18:
Line 405-411: There seems to be a sharp drop between Level E and Level F. E is interpretable but less acceptable than D (not unacceptable or ungrammatical), whereas F is uninterpretable and unacceptable (which presumably means the same as completely unacceptable and/or ungrammatical).

P. 19:
Line 419: "… no full translation of the whole sentence to English." ➔ "… no full translation of the whole sentence into English."

Line 422:
"The experiment comprised six blocks of 372 items." ➔
"The experiment comprised **<u>of</u>** six blocks of 372 items **<u>in total</u>**."

Line 424-425: Explain what is meant by "counterbalancing scheme".
Line 425: "… ensured that all items were seen equally often across the six blocks…"
Don't you mean that each type was used equally often and each item was seen only once?

Line 426-427: "…and particularly important when the number of items per block is higher than 1, …" I don't understand. Please explain. Clearly the number of items in each block is higher than 1. Otherwise, there would be 372 blocks…

Line 428: "Also target sentences were seen equally often in the four conditions." Do you mean "target sentence types"?

Line 430: I am not familiar with the term "Williams design". Please provide a reference.

Line 433-434: "This design requires a multiple of 24 subjects and determined the total number of 48 subjects." How can this "determine" the number of subjects? It seems that it requires at least 24 participants, and that you doubled the number to be sure.

Line 436: "After providing informed consent and collection of demographic information…" Which demographic information?

Line 438-439: Participants were asked to "to use the full spectrum of the seven-point scale". How was this done?

P. 20-21, "Statistical analysis"
This section needs to be significantly revised. It is almost impossible to follow.
Line 454: What is a "Helmert contrast"? Explain and add reference.

Line 459: "We selected a complex mixed model…" What do you mean? The full model? Please specify.

Line 461-467: "… we started with an LMM including VCs and PCs ..." This entire paragraph is opaque. What exactly was the model? What is meant by "at the boundary"? What do you mean "forcing CPs to zero"?

P. 21:
Line 477: "As the six types of filler sentences varied between items, …" Please explain.

Line 479-480: Please explain and add references for "Forcing CPs to zero", "AIC", and "BIC criteria".

Line 485-485: Please provide a reference for this.

P. 22:
Figure 1 (and the same applies to figures 2 and 3): It would be very helpful with standard asterisks indicating significant contrasts (satiation effects, horizontally), (*p<.05, **p<.001) (as well as vertically between types). Extracting this information from the text is in no way

easy (and it's not clear that it's all there). Also, there should be error bars (e.g., ±1 SE). I would also be helpful if the target sentences were labelled "SO aa", "SO ai", "OS ia", and "OS aa" (or similar) to better reflect the word order contrast and the contrast in animacy.

Line 501-504: It would be helpful to see the p-values as well.

P. 23:
Line 510-511: "As is clearly visible in the figure, ratings increased less strongly for "wer-wen…"" It seems to be zero, so "less strongly" is misleading.

Line 521-522: "The condition with mismatched animacy is almost on par with subject-initial conditions, …"
This seems to be speculation. In fig. 1b, "was_wer" (OS ia) is closer to the lower-ranking "wen_wer" (OS aa) than to the higher-ranking "wer_was" (SO ai) and "wer_wen" (SO aa). Again, visual indication of significant contrasts in the figures would be helpful.

P. 23-24:
Line 532-534: I don't understand. Please rephrase.

Line 543: "…there was no interpretable change …" Do you mean "significant"?

Line 545-546: "Figure 1b exhibits the corresponding pattern of mean changes between block 1 and block 2." ➔ "… between block 1 and **<u>the mean of blocks 2-6</u>**."

P. 25:
Line 562-563: "For the two object-initial targets we observe higher rating (b=0.40, z=4.24) for "was_wer" than "was_wen", …" The "was_wen" condition is subject-initial.

Line 566-567: "Possibly, D represents a "hard-core" ungrammatical filler type from the beginning" Do you mean "F", not "D"? If D is "hard-core" ungrammatical, then how come E behave like a less hard-core type?

Line 573-574: "… and (iii) exposure effects may be a property of intermediate judgements rather than of certain types of syntactic constructions."
Yes, but see type F. It does not show increased acceptability over time, and it is only example which is starkly ungrammatical. All the other fillers are increasingly unacceptable, but F is not just less acceptable than E. So, the results here are compatible with the assumption that fully acceptable sentence will not show "satiation", as they can't get any better, and fully ungrammatical sentences remain ungrammatical. Sentences with intermediate (degraded) acceptability are susceptible to satiation. This is also how I understand refs. [15] and [25], at least.

P. 26:
Line 599: Did you make sure that the participants were native speakers of English?
P. 28-29
Lines 644-666: The sentences in each level are not minimally different. Therefore, it is not appropriate to average across them and speculate on the interpretation of the contrasts, between filler types as well as (and more importantly) between targets and fillers. The only thing which does allow for interpretation is that some of them show a satiation effect. That says nothing about the status of the well-formedness and satiability of the target sentences.

P. 29:
Line 674: Explain “counterbalancing”.
Line 680: Explain how participants were instructed to use the full 7-point scale.

P. 31:
Fig. 2: See my comments to fig 1. above.

Line 707-710: Are you talking about an overall average (across all types) or about onbe of the types?

P. 32:
Line 722: “… (see Figure 2)” ➔ “… (see Figure 2**b**)”

Line 723:
What is meant by “the prototype pattern”?

Line 724-726: These stats are different from the ones in line 713-714. Am I missing something?

Line 729: “respectively”?

Line 729-730: What is meant by “statistically parallel”?

Line 735:
“In summary, as for German target sentences, there is …” ➔
“In summary, in English target sentences, just like in the German experiment, there is …”

P. 33:
Line 745: “We chose to run the Experiment 2 …” ➔ “We chose to run Experiment 2 …”

Line 783: Explain “use the full spectrum of the seven-point scale”.

P. 35
See my comments about fig. 1 above.

P. 36:
Line 830-836: The numbering is wrong. It should be a-d, not g-j.

Line 831: “A shift in **the** intermediate zone…” So, do you take “intermediate to mean $1<x<7$ or $2<x<6$? None of the types are either 1 or 7…

P. 37:
Line 838: “… violations with like animacy …”?

Line 850:
“Our experiments provide strong evidence for an increase, …”
“Our experiments provide strong evidence for an increase in mean acceptability rating, …”

Line 852: “we have 12 per block, i.e. 48 target items per block.” I don’t understand. Please rephrase.

Line 853-854: “Previous studies vary between 5 and 7 blocks, each with 1 instance of the crucial constructions (some studies contain multiple islands (up to 6 in total) …”
What do you mean “Previous studies vary between 5 and 7 blocks”? What do you mean “some studies contain multiple islands (up to 6 in total)”?

Line 857: “… the results in the literature on the other hand are zooming in just on the initial part of the experiment (the first two blocks).”
This also applies to the present paper. Almost the entire discussion is based on fig. 1b, fig. 2b, and fig. 3b., which all show the difference between block 1 and the mean value for blocks 2-6 – in effect, the first two blocks. If the point is that there is little or no significant change after block 2, then please make it explicit. Or am I missing something here?

P. 37-38:
Line 858-867: “First, one would like to know if repeated exposure eliminates the perception of non-well-formedness for certain constructions and for certain grammatical problems, or whether the penalty for violating certain laws of language is merely mitigated. The first interpretation may suggest itself if a certain violation is thought to be a result of the type of processing difficulty proposed in [18] that should no longer exist after a sufficient amount of exposure. In contrast, the second interpretation would be a natural consequence of the view developed in [20] for instance, according to which satiation effects result from improved comprehension strategies (while the perception of grammatical problems would be left intact).”

First, repeated exposure does not necessarily “eliminate the perception of non-well-formedness” such that the “processing difficulty” associated with “no longer exist after a sufficient amount of exposure”. Rather, what seems to be argued in [18] as well as in [25] and others is that processing difficulty can be reduced. It doesn’t have to be eliminated. In fact, it is not clear to me why it should be. For example, if the heavy load on working memory can be mitigated with repeated exposure, say with center-embeddings or with certain types of island violations, it doesn’t follow that the participants in the experiments would get massively improved syntactic working memory capacities. It might be improved to a certain point, though (as with other types of memory training). Maybe this is a middle way between the two “interpretations” presented here. The second interpretation, I think, may also be too strong. I agree that there may be alternative or “improved comprehension strategies” involved, but I don’t think that “the perception of grammatical problems” need be “left intact”, because there may not be any “grammatical problems”. Perhaps the reduced / intermediate acceptability rating reflects processing difficulty, as is indeed the case for unambiguously grammatical long-extraction (e.g., crossing two or more clause boundaries) and arguably for certain island violations [25].

Page 38-39
Line 883-890: “… by looking at where response times destabilise. …. From a visual examination of the log-transformed response times…. Notably, response times stabilised…”
You have not included response time measurements in results. (And for experiments 1 and 2, it is not clear how it could have been measured.)

Line 905: “The hope in some of the generative literature [8] that repeated exposure may make questionable sentences (nearly) completely acceptable may be unjustified in any event.”
As in my comment for line 858-867, I think this is too strong. The idea in the literature is not (necessarily) that “questionable sentences” (with reduced acceptability) can be made “(nearly) completely acceptable”. Only that their acceptability can be improved over time. Furthermore, I don’t think that the word “hope” is appropriate here.

P. 40:
Line 921: Again, I think the word “hope” is inappropriate.

Line 927-928 & 929-930: Please specify what is meant by a “range of judgments”.

Line 935-936: “So, we observe upward movements of well-formedness for items that may have an unusual object choice, crossing movement, and an ordering constraint. There is no obvious common factor to these.”
There doesn’t have to be any “obvious common factor to these”. These sentences are all fillers and (as far as has been described in the paper, at least) you did not control for any of the factors you mention here. You conflate and average across different sentences types (and violation types) to get different acceptability ratings along the scale, which makes actual comparison between filler levels and between filler and targets inappropriate.

Line 937-942: The same applies to English. Indeed, verb-object adjacency can be sometimes be violated, and sometimes it must be violated (‘I saw _ yesterday what might be the ugliest building in the World’), and it’s conceivable that perceived intonation of deviant sentences might facilitate a ‘heavy NP-shift’ reading.
