# Supplementary material for: Middle ratings rise regardless of grammatical construction: Testing syntactic variability in a repeated exposure paradigm
Source: PLoS One. 2021 May 11;16(5):e0251280. doi: 10.1371/journal.pone.0251280 (PMC8112649; doi:10.1371/journal.pone.0251280)
Supplement: Attachment — Submitted filename: BFHK-3-response.docx [file pone.0251280.s009.docx]

**Reply to reviewer’s comments**

**Major comment:**

- Comment:

It would be nice if you could add black dotted horizontal lines to indicate the satiation zone to the plots in Fig1b, Fig2b, and Fig3b. It would also be very helpful if you would add an asterisk for example to the immediate right of the lines for significant changes in Fig1b, Fig2b, and Fig3b. These small additions would be easy to make and would not clutter the plots, and they would make it much easier to see what the plots actually show – both in terms of significant effects (vs. mere trends) as well as differences between English and German in the range of the intermediate zone of satiation.

*Reply*: We very much appreciate this proposal, changed the figures accordingly, and agree that it substantially improves what we want to communicate with them. The adoption of this proposal necessitated a change in the specification of the second LMM for each experiment such that the change from block 1 to the average of block 2 to 6 is tested with block nested within levels of sentence type, not as crossed with contrasts defined for types. These statistics are definitely much more comprehensible than the prior ones relating to interactions between contrast and block because with this parameterization asterisks in the figures correspond directly to estimates in the post-hoc LMM. As a side effect we also corrected an error in the labelling of types with a very similar profile of acceptability in the figure of Experiment 3. We note that this was not our initial plan for data analysis, but we consider it acceptable to switch from one post-hoc LMM to a different one. This change required changes in the methods and results. We also include tables with fixed-effects in the supporting material. Again, thank you very much for this comment!

**Minor comments:**

- Comment:

L.23-24:
“A prominent approach in linguistic theory claims that” =>
“A prominent approach in linguistic theory argues that”

*Reply*: changed

- Comment:
  L.32-33:
  “Second, ratings asymptote below maximum acceptability, that is they do not satiate.” =>
  “Second, though there is satiation, ratings asymptote below maximum acceptability.”
  As is also argued in the paper, the increase in acceptability as a function of exposure IS satiation, and satiation does not necessarily need to lead to full acceptability.

*Reply*: changed

- Comment:
  L.67:
  “fully inacceptable” =>
  “fully unacceptable”

*Reply*: changed

- Comment:
  L.98
  “illustrated in (1). in which” =>
  “illustrated in (1) in which”

*Reply*: changed

- Comment:
  L.121, table 1 (Goodall (2011), Adjunct island):
  “nos” => “no”

*Reply*: changed

- Comment:
  L.156:
  “the Complex Noun Phrase Constraint. (1e) violations” =>
  “the Complex Noun Phrase Constraint (1e) violations”

*Reply*: changed

- Comment:
  L.157:
  “not, e.g., that-trace violations 1c)” =>
  “not, e.g., that-trace violations (1c))”

*Reply*: changed

- Comment:
  L.175:
  “Goodall [14] provides another example for the sensitivity” =>
  “Goodall [14] provides another example of the sensitivity”

*Reply*: changed

- Comment:
  L.182:
  “The variability between experiments visible calls…” =>
  “The variability between experiments calls…” =>

*Reply*: changed

- Comment:
  L.218, (2b):
  Use a trace (t) like in (1) instead of strikethrough.

*Reply*: changed

- Comment:
  L.223:
  [22] => [23]

*Reply*: changed

- Comment:
  L.258:
  “Examples for the lower three levels of English”
  Please explain the scale, what the levels (C,D,E) mean. The scale is not explained in detail until lines 376-382, where it actually also explained that an extra level is introduced (F). So “lower” is not the right term here.

*Reply*: changed (changed “lower” to “intermediate English levels”)

- Comment:
  L.274:
  “because German material is” =>
  “because the German material is”

*Reply*: changed

- Comment:
  L.277:
  “because of the sentence does not respect” =>
  “because the sentence does not respect”

*Reply*: changed

- Comment:
  L.342-3:
  “(A) "almost not well-formed" to (E) "completely well-formed".”
  “(A) "completely well-formed" to (E) " almost not well-formed ".”

*Reply*: changed

- Comment:
  L.360:
  “[31] design” =>
  “design [31]”

*Reply*: changed

- Comment:
  L.385:
  “The material consisted of 120 targets …” =>
  “The material consisted of 120 target quadruples (corresponding to (3) above)” …

*Reply*: changed

- Comment:
  L.385:
  “… and 252 fillers. that is the ratio of targets” =>
  “…and 252 fillers. That is, the ratio of targets”

*Reply*: changed

- Comment:
  L.447:
  “Williams [31] design” =>
  “Williams design [31]”

*Reply*: changed

- Comment:
  L.501:
  “block 2 did not differ (C1 to C5 [39].” =>
  “block 2 did not differ (C1 to C5) [39].”
  What is “C1 to C5”? Presumably “C1 to C5” refers to “five of these nine block x type-of-sentences contrasts”, but that is not clear. Since, the details have been moved to the OSF repository. This bit should be left out here. Also, it’s not clear why we need a reference to [39] here.

*Reply*: C1 to C5 referred to contrasts defined for sentence types. They are no longer the part of the post-hoc LMM where we test block as nested within type of sentence.

- Comment:
  532-4:
  “As is clearly visible in Fig 1, ratings increased less strongly for “wer-wen” and more strongly for “wer-was” constructions than the other three conditions.”

  It is NOT “clear”. It looks very much as if “wer_was” and “wen_wer” increase equally much.

*Reply*: the reviewer mentions “wer_was” where we mentioned “wer-wen”. We think the misunderstanding may come from the fact that wer-wen does not increase at all. We were intending to highlight the differences between conditions here (i.e., the contrasts). We no longer have a test of this interaction contrasting “wer_wen” with the *average* of the other three conditions. The simple tests yielded there is no change for “wer_wen”, but significant changes for the three other types of target conditions.

- Comment:
  L.616:
  “generative literature.” =>
  “generative literature).”

*Reply*: changed

- Comment:
  L.621 & L.627:
  “non-discourse-linked object” =>
  “non-discourse-linked wh-phrases”
  Both subject and object are non-D-linked.

*Reply*: changed

- Comment:
  L.757:
  “(both z<1.).” =>
  “(both |z|<1).”

*Reply*: changed

- Comment:
  L.854-5:
  “Just as in the preceding two experiments, filler level F behaved in an unpredicted way by not showing any signs of rising judgments.”
  Unless I’m missing something, the behavior of F is not unexpected. Since it is tailored to be fully ungrammatical and hence be below the intermediate zone, it is predicted to not show satiation. It’s the behavior of E that is unexpected.

*Reply*: We agree with this comment. In the Discussion we clarify that the rise of E most likely relates to a delayed recognition of less severe ungrammaticality than F (as intended). This should not be confused with a rise of acceptability of these sentences; they are still judged ungrammatical.

- Comment:
  L.948:
  “Zajonc [46]” =>
  “Zajonc [45]”

*Reply*: changed

- Comment:
  L.961
  “Zajonc [46]” =>
  “Zajonc [45]”

*Reply*: changed

- Comment:
  L.962:
  “Häussler [47]” =>
  “Häussler [46]”

*Reply*: changed

- Comment:
  L.970:
  “Sprouse et al., [48]” =>
  “Sprouse et al., [47]”

*Reply*: changed

- Comment:

L.974:
“variants of targets However, the fillers” =>
“variants of targets. However, the fillers”

*Reply*: changed
